# High prevalence of metabolic syndrome in a mestizo group of adult patients with primary hyperparathyroidism (PHPT)

**DOI:** 10.1186/s12902-015-0014-5

**Published:** 2015-04-03

**Authors:** Victoria Mendoza-Zubieta, Gloria A Gonzalez-Villaseñor, Guadalupe Vargas-Ortega, Baldomero Gonzalez, Claudia Ramirez-Renteria, Moises Mercado, Mario A Molina-Ayala, Aldo Ferreira-Hermosillo

**Affiliations:** Endocrinology Departament Hospital de Especialidades Centro Médico Nacional Siglo XXI, Instituto Mexicano del Seguro Social (IMSS), Cuauhtemoc N° 330, Colonia Doctores, México City, DF Mexico; Endocrinology Experimental Investigation Unit Hospital de Especialidades Centro Médico Nacional Siglo XXI, Instituto Mexicano del Seguro Social (IMSS), Cuauhtemoc N° 330, Colonia Doctores, México City, DF Mexico

**Keywords:** Primary hyperparathyroidism, Metabolic syndrome, Central obesity, Parathyroid hormone

## Abstract

**Background:**

Primary hyperparathyroidism (PHPT) and metabolic syndrome (MS) have been independently related to cardiovascular morbidities, however this association is still controversial. Mexican population has a high prevalence of metabolic syndrome, however its frequency seems to be even higher than expected in patients with PHPT.

**Methods:**

We retrospectively reviewed the charts of patients that underwent parathyroidectomy for PHPT in a referral center and used the criteria from the National Cholesterol Educational Program (NCEP)/Adult Treatment Panel III (ATP III) to define MS before surgery. We compared the characteristics between the patients with and without MS.

**Results:**

60 patients were analyzed, 77% were female and 72% had a single parathyroid adenoma. MS was present in 59% of the patients, this group was significantly older (57 vs. 48 years, p = 0.01) and they had lower iPTH (115 vs. 161 ng/ml, p = 0.017). Other parameters did not show differences.

**Conclusions:**

MS is frequent in our population diagnosed with primary hyperparathyroidism, adverse cardiovascular parameters are common and significant differences in calcium metabolism compared to the non-MS group are present.

## Background

Primary hyperparathyroidism (PHPT) is a common endocrine disease, particularly in postmenopausal women. It is the main cause of hypercalcemia in outpatient settings and its prevalence is estimated to be 1 to 4 per 1000 people in the general population and 21 per 1000 in postmenopausal women [1]. The estimated incidence is 15.7/100.000 people /year in the USA [2].

PHPT is also associated with an increased risk of cardiovascular mortality [3-5]. Recent evidence shows that it is also associated with metabolic disorders and other components of metabolic syndrome (MS) such as hypertension (HTN), dyslipidemia, glucose intolerance, obesity, insulin resistance and reduced insulin secretion [6-8]. The ethiopathogenic mechanisms of MS are not completely understood and the association with calcium metabolism is being studied, since calcium is essential for many metabolic processes. Additionally, changes in vitamin D levels and other hormones in obese populations and patients with PHPT could be involved in the pathogenesis of cardiovascular and metabolic comorbidities. The association between these entities is complex and may be a source of additional morbidity and mortality.

The prevalence of MS, obesity and insulin resistance have increased continuously all around the world. Each one of them have been well documented as causes of increased cardiovascular morbidity and mortality [9-11]. However, the prevalence of MS varies according to definition criteria, age, sex and population studied. Between 1999–2010 the National Health and Nutrition Examination Survey (NAHNES) in USA reported an accumulated prevalence of 25.5% in adults older than 20 years of age; this prevalence decreased to 22.9% in 2010 [12]. In Europe the Diabetes Epidemiology: Collaborative Analysis of Diagnostic Criteria in Europe (DECODE) reported a prevalence of MS of 41% for male population and 38% for female population according to International Diabetes Federation (IDF) criteria [13]. In Mexico, the prevalence in adults older than 20 years of age is 41.6% according to the criteria proposed by the American Heart Association/National Heart Blood and Lung Institute (AHA/NHLBI) and 42% using the (NCEP:ATPIII) criteria [14]. Some polymorphisms associated altered lipid metabolism pathways are frequent in Mexico, which may be associated with the higher prevalence of MS in this mestizo population [15,16], however, other components of the MS are also frequent in these population. These data suggests that MS is twice as prevalent in our population compared to other countries, which also may have an influence on the calcium and vitamin D metabolism in both physiological and pathological states. The ethiopathogenic relationship between PHPT and MS is currently being investigated, it‘s unclear if the association of these two entities will affect mortality in the long term. Since Hispanic populations seem to be especially prone to high risk profiles for cardiovascular disease, specific analysis of these groups may help determine which patients require special diagnostic or treatment algorithms.

The purpose of this study was to evaluate the prevalence of MS in patients with PHPT and to compare the clinical and biochemical characteristics of patients with and without MS.

## Methods

This cross-sectional study included patients from the Bone and Calcium Metabolism Clinic of the Hospital de Especialidades Centro Medico Nacional Siglo XXI diagnosed with PHPT during a 3 year-period between 2006 and 2009. A general evaluation including anthropometry was performed in all patients before surgery. We evaluated the signs and symptoms related to hypercalcemia (repeated nephrolithiasis, gastritis, polyuria, muscle weakness, osteosteoporosis or psychiatric disorders), personal history of bone fractures and we assessed biochemically for hypercalcemia, high blood level of intact parathyroid hormone (iPTH) and hypophosphatemia. Nephrolithiasis diagnosis was assessed through kidney ultrasonography only in patients with related symptoms. We also performed a bone densitometry through DEXA (dual-energy X-ray absorptiometry) to evaluate osteopenia or osteoporosis. We performed calcium:creatinine clearance ratio only in asymptomatic patients with a family history of hypercalcemia in order to differentiate between PHPT and familial hypocalciuric hypercalcemia (data not shown). Patients were subjected to localization studies such as neck ultrasonography and Tc99-sestamibi scintigraphy. Only the patients with confirmed PHPT (symptomatic or asymptomatic), which had an indication for surgery (serum calcium > 1.0 mg/dl above upper limit of normal range, BMD T score ≤ −2.5 at any site or fragility fracture, calculated glomerular filtration rate below 60 ml/min and age ≤ 50 years, according the Third International Workshop [17]), were included in the final analysis. After surgery, histopathology report was also reviewed. Subjects with incomplete clinical, pathology or biochemical evaluation were eliminated. Sixty patients fulfilled these criteria and were included for analysis. Information about demographic characteristics, previous diseases as hypertension, impairment of glucose metabolism (defined as impaired glucose tolerance in an oral glucose tolerance test or impaired fasting glucose) T2DM, dyslipidemia or obesity, diet and exercise habits were obtained from medical records.

The study was performed according to Declaration of Helsinki II and completed all the requirements of the local ethics committee (Comité Local de Investigación y Ética en Investigación en Salud). The protocol and the aim of the study were fully explained to the subjects, who gave their written consent.

### Diagnostic criteria of the metabolic syndrome

Metabolic syndrome was defined according to the NCEP:ATPIII criteria as the presence of three or more of following components: waist circumference (WC) ≥102 cm in men and 88 cm in women, triglycerides > 150 mg/dl, c-HDL < 40 mg/dl in men and <50 mg/dl in women or a previously treated dyslipidemia, Systolic Blood Pressure of 130 mmHg or Diastolic Blood Pressure of 85 mm Hg or physician’s diagnosis of hypertension or patients receiving treatment with antihypertensive drugs and fasting glucose >100 mg/dl or physician’s diagnosis of diabetes [18].

#### Anthropometric measurements

At the time of evaluation we registered weight (kilograms) and height (meters), as well as waist circumference [WC] (centimeters). A single investigator, using the same calibrated scale with an integrated stadiometer, performed all the anthropometric measurements. WC was determined at the middle point between the inferior rim of the last costal arch and the superior rim of the anterosuperior iliac spine. Central obesity was defined using WC as previously mentioned. Body mass index (BMI) was calculated as the weight divided by height to the square. Those patients with BMI between 25–29.9 kg/m^2^ were considered overweight and the ones with a BMI ≥30 kg/m^2^ were considered obese according to World Health Organization (WHO) criteria. Blood pressure was determined in the left arm, after 10 minutes in a resting position, during a fasting state, without coffee or tobacco ingestion in the last week. The sphygmomanometer was calibrated and values were averaged after 2 different measurements with a 5-minute difference between them.

#### Biochemical determinations

For biochemical determinations all patients fulfilled a fasting state of 12 hours. Laboratory results were obtained with a 6 mL sample in BD Vacutainer (BD Franklin Lakes, New Jersey USA), centrifuged at 3150 *x g* for 15 minutes and serum was then analyzed with a kit for glucose, cholesterol, c-HDL and triglycerides (COBAS 2010 Roche Diagnostics, Indianapolis USA) using photocolorimetry with spectrophotometer Roche Modular P800 (2010 Roche Diagnostics, Indianapolis USA). c-HDL samples were treated with enzymes modified with polyethileneglycol and dextrane sulphate, analyzed with the same photocolorimetric technique. We used a specific chemioluminiscent assay to measure iPTH (DiaSorin Inc, Stillwater, Minnesota) with a sensitivity of 1 pg/ml and inter- and intra-assay coefficient of variation (CV) of 5.3% and 3.5%, respectively. Serum calcium (Ca) and phosphate (P) were tested using automated methods based on colorimetric and enzymatic assays (COBAS, Roche).

#### Statistical analysis

Results are expressed according to sample distribution with means and standard deviations (SD) or medians and interquartile ranges (IQR). Kolmogorov-Smirnov test was used to determine normality. To establish associations between quantitative variables, Student’s *t* test or Mann–Whitney *U* test were used. Qualitative variables were associated with χ^2^ or Fisher tests. Correlations between quantitative variables were tested with Pearson or Spearman test. A *p* < 0.05 was considered to be significant. Data were analyzed with SPSS v.16.

## Results

### Anthropometrical and clinical characteristics

Sixty patients with an average age of 53 years, completed clinical and biochemical assessment: 46 female and 14 male with a proportion of 3:1. According to our data, 65% of patients have a family history for T2DM and 48.3% for hypertension (data not shown). We found that 60% of patients (n = 36) had impaired glucose metabolism, 30% of patients had T2DM (n = 18), 68% (n = 41) had hypertriglyceridemia, 62% (n = 37) had central obesity, 40% (n = 24) had hypertension and 52% (n = 31) had low c-HDL concentration. In total, 60% (n = 36) of the patients with PHPT fulfilled MS diagnostic criteria according to the NCEP:ATPIII definition. Baseline characteristics of population are referred in Table 1.

**Table 1:** **Baseline characteristics of patients with PHPT**

**Parameter**	**n = 60**
**Age (years), mean ± SD**	53.5 ± 13.9
**Female (%)**	77
**Weight (kg), median (IR)**	68.2 (61.1-81.4)
**Height (m), mean ± SD**	1.57 ± 0.09
**BMI (kg/m** ^**2**^ **), mean ± SD**	29.08 ± 4.9
**Waist (cm), median (IR)**	91.5 (82.5-106.5)
Female	90.5 (83.5-105.5)
Male	97 (82–109)
**Glucose (mg/dl), median (IR)**	103 (87–115)
**Calcium (mg/dl), mean ± SD**	11.1 ± 1.09
**Phosphorus (mg/dl), mean ± SD**	3.08 ± 0.68
**iPTH (ng/ml), median (RI)**	127.5 (84.5-185)
**Triglycerides (mg/dl), median (IR)**	189.5 (130–269)
**c-HDL (mg/dl), mean ± SD**	47.3 ± 11.8
Female	48.7 ± 12.27
Male	42.7 ± 9.23
**Clinical manifestations**	
Pancreatitis	1%
Osteopenia	33%
Gastritis	37%
Osteoporosis	45%
Nephrolithiasis	52%
Muscular weakness	58%
**Histopathology report**	
Cancer	2%
Double adenoma	3%
Hyperplasia	23%
Single adenoma	72%

*Abbreviations*: BMI = body mass index, iPTH = intact parathyroid hormone, SD = standard deviation, IR = interquartile range.

When we compared patients with PHPT and MS (n = 36) and without MS (n = 24), we found that 89% had hypertriglyceridemia, 78% had alterations of glucose metabolism, 44% had T2DM, 83% had central obesity (according to sex), 58% had hypertension and 64% had low c-HDL concentration. In the group of patients that did not fulfill the MS criteria 29% (n = 7) had central obesity, 13% (n = 3) had HTN, 38% (n = 9) had hypertriglyceridemia, 33% (n = 8) had low c-HDL concentration, 33% (n = 8) had alterations on glucose metabolism and 8% (n = 2) had T2DM. It is noteworthy that not one of the patients in this study had completely normal metabolic parameters. Table 2 depicts other clinical and biochemical differences between groups. Considering that the age between groups was statistically different, we performed a stratified analysis dividing patients by age and sex. Most clinical and laboratory parameters were similar between these groups except for hypertension. Hypertensive patients were significantly older than the normotensive ones in both genders (66 + 20.2 years of age vs. 43 + 15.9, p = 0.037 for males and 60.7 + 9.8 vs. 49.7 + 10.7, p = 0.001 for females).

**Table 2:** **Baseline clinical and biochemical characteristics of patients with PHPT with and without MS**

	**With MS**	**Without MS**	***p***
**Age (y), mean ± SD**	57.2 ± 12.6	48 ± 14.2	0.014^a^
**Female, n (%)**	27 (75)	19 (79)	0.78
**Weight (kg), median (IR)**	73 (63–85)	63.5 (54–71)	0.010^b^
**Height (m), mean ± SD**	1.57 ± 0.09	1.56 ± 0.09	0.65
**BMI (kg/m** ^**2**^ **), mean ± SD**	30.3 ± 4.58	27.1 ± 5.02	0.017^a^
**Glucose (mg/dl), median (IR)**	107 (96.7-126.5)	90 (84.5-102.7)	0.006^b^
**Calcium (mg/dl), mean ± SD**	10.8 ± 0.93	11.43 ± 1.25	0.07^a^
**Phosphorus (mg/dl), mean ± SD**	3.2 ± 0.60	2.9 ± 0.77	0.114
**iPTH (ng/ml), median (IR)**	115 (73.6-154.2)	161.5 (100.9-231.5)	0.017^b^
**Triglycerides (mg/dl), median (IR)**	231.5 (168.7-289.5)	133 (113.7-178.2)	<0.001^b^
**c-HDL (mg/dl), mean ± SD**			
**Female**			
**Male**	46.19 ± 12.6	52.42 ± 11.08	0.090
	41.78 ± 11.14	44.6 ± 4.72	0.604
**Central obesity, n (%)**	30(83)	7(29)	<0.001^c^
**Hypertension, n(%)**	21(58)	3(12.5)	<0.001^c^
**Hypertriglyceridemia, n(%)**	32(89)	9(38)	<0.001^c^
**Low c-HDL concentration (%)**	23(64)	8(33)	0.019^c^
**Impairment of glucose metabolism, n(%)**	28(78)	8(33)	0.001^c^
**T2DM, n(%)**	16(44)	2(8)	0.002^c^

*Abbreviations*: BMI = body mass index, T2DM = type 2 diabetes mellitus, IR = interquartile range, iPTH = intact parathyroid hormone. ^a^Student *t* test, ^b^Mann–Whitney *U* test, ^c^Fisher’s exact test.

We also found that 37% of total population was overweight and 33% were obese (according to BMI). In patients with MS the prevalence of obesity increased to 39% while overweight was present in 47%. In patients without MS the prevalence of excess weight and obesity was 21% and 25%, respectively; also, 87% of them had at least one component of MS.

We found that iPTH levels were higher in patients without MS (Table 2). Despite levels were not significant between groups, we found that phosphorus levels were negatively correlated with iPTH (ρ = −0.416, p = 0.001) and that calcium levels were higher in patients without MS. We did not find any correlation between iPTH, calcium and other metabolic parameters (data not shown).

## Discussion

Our study shows that Mexican patients with PHPT have the highest prevalence of MS reported so far, reaching a staggering 60% of the cases. However, we should note that MS prevalence varies widely depending on diagnostic criteria used and the population studied. Nonetheless, other authors using the same criteria (NCEP:ATPIII criteria) reported lower prevalences; deLuis et al. reported a prevalence of MS of 32.3% in 62 patients with PHPT from Spain [19], Procopio et al. [20] found a prevalence of 47.6% in patients classified as low-risk asymptomatic, 8.7% in symptomatic patients and 8.3% in high-risk asymptomatic patients and Luigi et al. [21] reported a prevalence of 38% in 30 patients from University of Rome “Sapienza”, Italy. In fact, according to our results, the presence of MS could be involved in the increased cardiovascular risk observed in patients with PHPT, which contrasts with Tassone et al. who reported that MS prevalence was similar between patients with PHPT and the general Italian population (Progetto Cuore Study) [22]. Unfortunately we don’t have enough information comparing symptomatic or asymptomatic patients to compare with this group. At this point, we should consider that Mexico has one of the highest prevalences of obesity and hypoalphalipoproteinemia in the world (76.3% according to national health survey “ENSANUT 2006”), which may bias the whole sample and artificially increase the overall prevalence of MS. However the prevalence of MS is even higher in the patients with PHPT than in the general population of Mexico (36% according to ENSANUT 2006) [14]. More studies are required to assess if the presence of MS is involved in the higher frequency of cardiovascular risk factors associated with PHPT.

MS components are related to insulin resistance [10]. In our country, recent national health survey “ENSANUT 2012” reported that the prevalence of overweight and obesity is almost 73% [23], which is consistent with the 70% in our present findings. It seems that fatty acids released from adipose tissue in obese patients causes insulin resistance through disruption in insulin signaling cascade [24], increased inflammation, oxidative stress, coagulation abnormalities, endothelial damage, myocardial dysfunction and accelerated atherosclerosis [10,11]. We found that central obesity is one of the most frequent components of MS in this group (83% in MS group vs. 29% in the non-MS group, p < 0.001), just second to hypertriglyceridemia. The high prevalence of central obesity in these patients could be explored as the major cause of cardiovascular risk.

There are certain molecular associations between adipose tissue and PHTP. Some in vitro experiments showed that the increased levels of PTH rises intracellular calcium inside the adipocyte, promoting an increase in its activity for as much as 50-100%, and an increase in the expression of fatty acid synthetase, increase in the activity of glycerol-3-phosphate dehydrogenase and inhibition of basal lipolysis [25]. These changes could be associated to the increased lipogenesis and an increase in adipocyte volume and could contribute to hypertriglyceridemia, which was higher than reported for general population (31.4%) [14]. On other hand, it has also been demonstrated that PTH stimulates the adipose tissue differentiation and increases insulin resistance in these cells [26] in correlation to the adipose tissue mass. Finally, some studies suggest that increased fat mass induced by the increased concentrations of PTH acts as a leptin mediator [27]. Nonetheless de-Luis et al. [19] found that serum levels of leptin and adiponectin are not related to iPTH, vitamin D or calcium levels in patients with PHPT. Further data is required to clarify the inflammation component of PHPT in MS.

Other studies correlate the increased mass of adipose tissue with the decrease in the 25-hidroxyvitamin D concentration as well and a secondary form of hyperparathyroidism [28]. This deficiency in vitamin D seems to be associated to abnormalities in the carbohydrate metabolism but pharmacological approaches haven’t shown favorable results [29]. Norenstedt et al. [30] studied 150 patients with PHPT after parathyroidectomy randomized in two groups, one with oral calcium carbonate and the other with calcium carbonate plus cholecalciferol. These authors found no improvement in metabolic parameters after one year of supplementation, despite the decrease in PTH levels. In the period where these patients were studied, vitamin D was not considered to be part of the diagnostic algorithm of PHPT and therefore was not available in our hospital. We consider that the low serum calcium reported in most of these patients, and even lower concentrations found in MS patients may have multiple causes. For once, it may be related to severe vitamin D deficiency, which is very frequent in the Mexican population (24% vs. 1% in other populations) [31] and has been reported to be even lower in obese patients [32]. The lower vitamin D concentrations usually seen in Mexican patients may have an impact in the calcium metabolism reported in our patients. Also, most of the patients were classified as having a relatively mild hyperparathyroidism, which is usually accompanied by lower serum calcium and PTH levels, unlike other reports were hyperparathyroidism was detected in more advanced stages. Other authors showed that obese patients with PHPT usually have higher PTHi levels than the ones in our report; however, all patients fulfilled diagnostic criteria for PHPT and had other indications for surgery, such as recurrent lithiasis or severe osteoporosis. Also, pathologic and imagenologic studies confirmed diagnosis. Clinical data associated with hyperparathyroidism may be an important consideration when a patient has a mildly altered laboratory results but damage to target organs such as bone or kidney are present.

Hyperparathyroidism (in any of its clinical presentations) is associated with altered carbohydrate metabolism and a disturbed insulin secretion or impaired cellular sensitivity [33,34]. In fact, Kumar et al. found that insulin sensitivity was lower in PHTP patients in comparison with a control group (60% vs. 113.7%) [35]. Procopio et al. found that almost 40% of patients with hyperparathyroidism have glucose intolerance in comparison with 25% found in healthy controls [33,34]. On the other hand, the prevalence of PHPT in patients with T2DM is 15%, (3 to 5 times higher than in patients without diabetes) [14]. According to data from ENSANUT 2012 the prevalence of T2DM in general population was 8.9% for patients between 40 to 49 years old and 19.2% for patients between 50 to 59 years old [36]. In our study, the prevalence of T2DM was 44%, twice than that reported for general population in any age group.

Regarding hypertension, we observed a prevalence of 40% in PHPT patients that is higher than in general population: 32.4% for male and 31.1% for female, [37,38]. A previous study has suggested that calcium levels are implicated in the pathogenesis of hypertension [39]. It seems that the increase in PTH levels promotes endothelial calcium influx and vasoconstriction. At this point, Petramala et al. found a positive correlation between iPTH levels and blood pressure [21]. However, in our study calcium and PTH levels were lower in patients with MS in comparison with patients without MS. This was also observed by Procopio et al. [20] and suggests that other metabolic a, such as vitamin D deficiency, could be involved.

Age and sex may be factors associated with the development of metabolic syndrome. In our study, the group with MS was statistically older than the group without it, which may reflect the evolution of MS trough time. Pairing patients by age or following them for several years, may reveal differences in the development of MS between patients treated surgically and the ones that haven’t, however, this study may not be ethical in patients with overt or severe PHPT. Long term follow up studies have not been conclusive in terms of determining the usefulness of treating PHPT in order to improve cardiovascular risk profiles, since most of them have been followed for periods of around 1 year after surgery, which is not enough to correct some biochemical abnormalities and other confounding factors like diet and exercise which have not been controlled.

There are some limitations in our study that should also be considered. First, we studied a relatively small sample of PHPT patients, however we intended to have only patients whose diagnosis was confirmed by pathology and that had completed screening protocols. Second, the cross-sectional design of the study prevents us from comparing this group with a healthy control group. However this study is the first to describe the prevalence of MS in Mexican patients with PHPT, and the results suggest that MS may be more prevalent in some groups than previously expected. Populations where cardiovascular risk factors are frequent could be adversely affected if PHPT is also associated. Prospective studies of this population including wider searches for metabolic, inflammatory and cardiovascular parameters are warranted in order to properly assess their cardiovascular risk before and after surgery.

## Conclusion

Our data shows the highest prevalence of MS in patients with PHPT ever reported. In our population, this could be related to the high prevalence of overweight and obesity. The most prevalent components of MS in PHPT are hypertriglyceridemia, central obesity and glucose metabolism impairment. These prevalences are even higher than reported in general population. We suggest that MS is involved in the cardiovascular risk associated to PHPT.

### Ethical standards

The study was performed according to Declaration of Helsinki II and completed all the requirements of the local ethics committee (Comité Local de Investigación y Ética en Investigación en Salud). The protocol and the aim of the study were fully explained to the subjects, who gave their written consent.
